# PPH AND BIOLOGICAL GLUE IN PATIENTS WITH HIGH RISK OF BLEEDING IN STAPLED HEMORRHOIDOPEXY

**DOI:** 10.1590/0102-6720201700020009

**Published:** 2017

**Authors:** Eduardo Henrique PIROLLA, Fernanda Junqueira Cesar PIROLLA, Felipe Piccarone Gonçalves RIBEIRO

**Affiliations:** 1 Spaulding/Massachusetts General Hospital Labs of Harvard Medical School, Boston, USA.

**Keywords:** Hemorrhoid, Liver cirrhosis, Stents, Clopidogrel.

## Abstract

**Background:**

Stapled hemorrhoidopexy is a common treatment for grade 3 hemorrhoids. Patients with conditions that increase the risk of bleeding, as cardiac stents usage with clopidogrel bissulfate and liver cirrhosis, should receive an extra care in surgical procedures due to the high risk of bleeding. For this reason and for patients with third degree hemorrhoids we propose the use of stapled hemorrhoidopexy followed by the use of biological glue.

**Aim:**

Assess surgical outcomes in patients with hemorrhoids and high risk of bleeding submitted to stapled hemorrhoidopexy followed by biological glue.

**Methods:**

Between 2005 and 2015, 22 patients were analyzed, in a retrospective cohort study.

**Results:**

From 22 patients submitted to stapled hemorrhoidopexy followed by the use of biological glue, only one (4.5%) presented bleeding in the surgical postoperative. Patients do not have any other complications and pain in the postoperative period. The median (IQR) operation duration was 55 (12) min and the median (IQR) length of hospital stay after surgery was 3 (2) days.

**Conclusion:**

Patients with high risk of bleeding submitted to stapled hemorrhoidopexy followed by the use of biological glue presented very low rates of bleeding in the postoperative period.

## RESUMO

**Racional:**

Procedimento para o prolapso hemorroidário é uma forma de tratamento comum para hemorróidas de grau 3. Pacientes que apresentem condições que aumentam o risco de sangramento, como o uso de stents cardíacos associado ao uso de clopidogrel e cirrose hepática, devem receber cuidado redobrado em procedimentos cirúrgicos, devido ao alto risco de sangramento. Por esta razão é aqui proposto o procedimento para prolapso hemorroidário seguido da aplicação de cola biológica em pacientes com hemorróida de grau 3.

**Objetivo:**

Avaliar os desfechos cirúrgicos em pacientes com hemorróida de grau 3 e alto risco de sangramento submetidos ao procedimento para o prolapso hemorroidário seguido da aplicação de cola biológica.

**Métodos:**

Entre 2005 e 2015, 22 pacientes foram analisados, em estudo coorte retrospectivo.

**Resultados:**

Dos 22 pacientes submetidos ao procedimento para o prolapso hemorroidário seguido pelo uso de cola biológica, apenas um (4.5%) apresentou sangramento no pós-operatório. Os pacientes não apresentaram nenhuma outra complicação ou dor no período pós-operatório. O tempo mediano da operação foi de 55 (12) min e a média do tempo de internação hospitalar foi de três (2) dias.

**Conclusão:**

Os pacientes com alto risco de sangramento submetidos ao procedimento de prolapso hemorroidário seguidos pela aplicação da cola biológica apresentaram baixa taxa de sangramento.

## INTRODUCTION

Hemorrhoidal disease is a frequent involvement since more than 50% of the population over 50 years old has experienced symptoms^3^ ^,^ ^4^. The most usual complications of hemorrhoids are heavy bleeding, chronic unremitting prolapse of mucosal tissue, strangulation, ulceration and thrombosis^2^ ^,^ ^5^.

 A widely treatment of hemorrhoids is procedure for prolapse hemorrhoids (PPH) or stapled hemorrhoidopexy^6^. One complication of this or other hemorrhoid treatments are postoperative hemorrhage^1^.

However, in patients with high risk of bleeding, as cirrhosis and cardiac patients with anticoagulation drugs, surgery can result in a larger hemorrhage complicating even more the patient condition. 

Therefore, to minimize hemorrhage in patients with high risk of bleeding submitted to surgery we propose the use of procedure for prolapse hemorrhoids together with biological glue. 

The aim of this study was to realize an observational retrospective cohort to assess the complications after stapled hemorrhoidopexy and biological glue.

## METHODS

### Study overview

Data were analyzed retrospectively between 2005 and 2015, from private practice in São Paulo, Brazil. Written informed consent was obtained from all subjects.

### Study question

The study was designed to explore the possibility of the absence of bleeding in the postoperative of PPH and biological glue in patients with high risk of bleeding.

### Inclusion criteria

Were included patients with third degree hemorrhoids associated with liver cirrhosis by hepatitis B or C, all of them classified as Child-Pugh C, therefore with a severe coagulation disorder, acting like a complete anticoagulated patient. Were also included patients with third degree hemorrhoids associated with stent and clopidogrel bisulfate usage. All patients using stents and Clopidogrel had an INR superior to 3, which represents a high risk of bleeding.

### Study design

Data was collected from private practice and was analyzed in a retrospective cohort.

### Procedures

**FIGURE 1:**
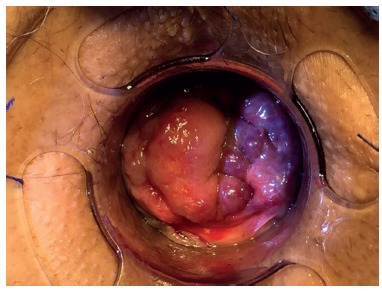
Third degree hemorrhoid

These 22 third degree hemorrhoids (Figure 1) patients underwent to the technique described by Longo, followed by the use of biological glue. All surgeries were performed by the same surgeon. 

**FIGURE 2:**
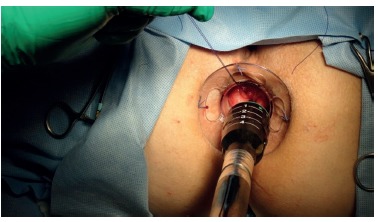
Insertion of circular stapler

**FIGURE 3:**
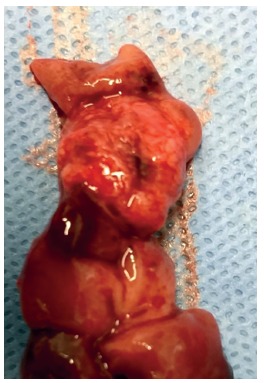
Surgical specimen after PPH

**FIGURE 4:**
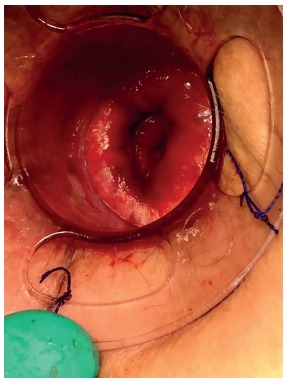
Result after using PPH

**FIGURE 5:**
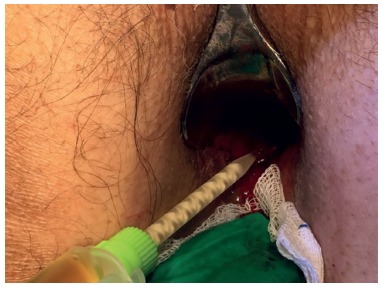
Use of biological glue in the suture line

All patients were submitted to clinical and laboratorial examinations before surgery. They underwent general anesthesia and were placed in lithotomy position. The circular anal dilator was inserted and sutured in the perineum. Purse string suture anoscope was then inserted and the purse string suture was performed above the dentate line and the anoscope was removed. Circular stapler (Figure 2) was then positioned and closed for about 60 s before fire. After the procedure surgical specimen was removed (Figure 3), suture line was observed (Figure 4) and biological glue was applied (Figure 5).

After the surgery feeding started on the same day, with high fiber diet and laxative diet, plus 2.5 liters of water. Analgesia was performed with acetaminophen and tramadol and if pain was bigger than 8, decimal solution of morphine sulfate was administered.

### Outcomes

Study primary outcome was the presence or absence of bleeding in the postoperative of PPH and biological glue. Bleeding was assessed by a nurse at each 2 h, until 3^th^ day of postoperative. Secondary outcomes were presence or absence of pain, which was also assessed by a nurse throughout a pain scale that ranges from 0 to 10 - 0 meaning no pain and 10 meaning the worse pain experienced. Pain was assessed every 6 h until day 3 of postoperative. Operation duration and length of hospital stay were also assessed.

### Statistical analysis

Categorical variables were analyzed with frequencies. Operation duration and length of hospital stay were continuous variables and were analyzed with median and interquartile range. Was used Small Stata Software, version 13 (StataCorp).

## RESULTS

### Patients demographic information

The study included 22 patients with third degree hemorrhoids associated with liver cirrhosis or clopidogrel use. Of the ten patients with liver cirrhosis five were due to hepatitis B and the other five to hepatitis C, all of them classified as Child-Pugh C, which represents a poor prognosis and an extremely high risk of bleeding. The other 12 patients were cardiac patients with the use of stents and daily use of Clopidogrel bisulfate, a platelet inhibitor. Patients using Clopidogrel bisulfate were with an INR higher than 3, which also represents a high risk of bleeding. Patients with liver cirrhosis and the ones using Clopidogrel bisulfate were predominantly male. In table 1 are represented the baseline characteristics of the 22 subjects submitted to PPH followed by biological glue application. 

**TABLE 1:** Baseline characteristics

	PPH + Biological glue (n=22)
Gender - n (%)	
Male	14 (64%)
Female	8 (36%)
Age - years (median - IQR)	58 (19)
Hemorrhoids grade III - no (%)	22 (100%)
Ethiology high risk of bleeding - no (%)	
Cirrhosis	10 (45%)
Clopidogrel bissulfate	12 (55%)

### Postoperative bleeding 

Of the 10 patients with hemorrhoids associated with hepatic cirrhosis none had bleeding during postoperative period. Of the 12 patients with hemorrhoid and stent, only one had bleeding during postoperative (Table 2). This patient presented bleeding (400 ml) during the first 24 h after surgery with no improvement after clinical treatment. This patient was submitted to a new surgery in the 3^rd^ day of postoperative, in which she was sutured with two stitches (Polydioxanone 3-0) in the stapled suture line with bleeding resolution (Table 3). Of the 22 patients only one presented bleeding in the postoperative period. Beyond this patient with bleeding there was no other complication reported in our cohort.

**TABLE 2:** Percentage of patients with bleeding after surgery

Bleeding	Presence	Absence
Cirrhosis (%)	0.0	100.0
Stent (%)	8.0	92.0
Total (%)	4.5	95.5

**TABLE 3:** Bleeding patient information

Blood loss	Gender	Age	Management	Outcome
400 ml	F	78	Reoperation- Two stitches in stapled suture line	bleeding resolution

### Pain and operation duration and hospital stay

All patient reported pain lower than 3 in a scale ranging from 0 to 10. The median (IQR) time of operation duration was 55 (12) min and patients stayed in hospital for a median (IQR) period of three 3 (2) days after surgery.

## DISCUSSION

Our results suggest that in patients with third degree hemorrhoids and liver cirrhosis or use of stent and Clopidogrel submitted to stapled hemorrhoidopexy followed by the use of biological glue has great results. Of the total cohort only one (4.5%) patient presented bleeding during the postoperative period. This represents a small percentage of the total sample. 

Searching in the literature was found two papers similar to our goal. The first one was a study from Anghelacopoulos et al. They did a randomized controlled trial comparing the use of PPH in one group and PPH and biological glue in the other. Their sample included patients with hemorrhoids grade 3 and 4, but without any condition that increase the risk of bleeding. They found results favoring the use of PPH followed by the use of biological glue. The second paper was from Huang et al. They have conducted a study that included patients with hemorrhoids and with liver cirrhosis submitted to PPH; however, they did not use biological glue; 25% of their sample presented bleeding after the procedure.

Despite the samples being a little different, our study shows encouraging results that resemble the results of Anghelacopoulos et al. Their study showed great results for a broad population while this one presents great results for a narrow population. 

Only with the use of PPH 25% of the sample presented bleeding after the procedure as reported by Huang et al. In our study with the use of PPH followed by biological glue 4.5% of our sample presented bleeding in the postoperative. Comparing only patients with cirrhosis our results presents 0% of postoperative bleeding against 25% in Huang et al. That suggests that biological glue associated with PPH might decrease postoperative bleeding in cirrhotic patients.

Based on our results, we propose that patients with third degree hemorrhoids with a secondary condition that increases the risk of bleeding should be submitted to stapled hemorrhoidopexy with the use of biological glue to reduce bleeding during the postoperative.

The association of third degree hemorrhoids and liver cirrhosis or stent use is not so frequent. For this reason, the major limitation of our paper is the reduced sample size. To achieve more solid and generalizable results one solution is the conduction of a multicenter prospective study to increase sample size. A prospective study could also help balancing the covariates between groups reducing confounders, that were not assessed in this study. But in surgical areas randomized controlled trial may face challenges as lack of infrastructure, surgeon learning curve and differences in development and research^7^. One factor that may have influenced our results were the great expertise of the surgeon.

## CONCLUSION

Patients with high risk of bleeding submitted to stapled hemorrhoidopexy followed by the use of biological glue presented very low rates of bleeding in the postoperative period.
